# Leveraging teachable moments in cancer prevention by improving HPV vaccination in health professional students (HPS): A systematic review

**DOI:** 10.3389/fonc.2022.978843

**Published:** 2022-08-29

**Authors:** Morgan S. Levy, Lindsey Finch, Kara A. Lindsay, Patricia Jeudin, Marilyn Huang

**Affiliations:** ^1^ Department of Medical Education, University of Miami Miller School of Medicine, Miami, FL, United States; ^2^ Department of Obstetrics, Gynecology, and Reproductive Sciences, Jackson Memorial Hospital, Miami, FL, United States; ^3^ Division of Gynecologic Oncology, Sylvester Comprehensive Cancer Center/University of Miami Miller School of Medicine, Miami, FL, United States

**Keywords:** HPV vaccination, cancer prevention, cervical cancer, medical education, interprofesssional education, vaccine hesistancy, public health

## Abstract

**Introduction:**

Vaccination against HPV is safe and effective in cancer prevention, yet vaccination uptake remains low. Strong recommendation of HPV vaccination by healthcare providers increases immunization rates, but gaps in knowledge persist surrounding HPV and HPV vaccination amongst health professional students (HPS). It is critical to educate HPS in all professions to maximize vaccination opportunities and increase vaccine uptake. The objective of this study is to evaluate evidence on HPV knowledge, vaccine uptake, and educational interventions in HPS to identify specific deficits to improve education.

**Methods:**

A systematic literature search for articles on HPV vaccine uptake, knowledge, and educational interventions in HPS was performed in PubMed, Embase, Web of Science, CINAHL, and Scopus from January 1, 2006 – July 21, 2021. Included studies assessed HPS for HPV vaccine uptake, knowledge, counseling comfort, or educational interventions to increase HPV vaccine knowledge. Studies were screened for inclusion by 2 independent reviewers and evaluated for risk of bias. PRISMA guidelines for reporting were followed.

**Results:**

Twenty-one unique articles met inclusion criteria and were included in the analysis. Of the studies included, 20 included knowledge, 11 included vaccine uptake, 8 included interventions, and 12 included counseling comfort. The students in the studies included medical (n=14), dental (n=7), dental hygiene (n=6), nursing (n=3), physician assistant (n=2), public health (n=1), and pharmacy (n=1). Across studies, HPV vaccine series initiation ranged from 34.6-70.3%, with 28.3-58.3% up to date on vaccination. Most students knew that HPV causes cervical cancer (99%), but fewer knew that HPV causes head and neck cancer (40-47%) and oropharyngeal cancer (45%). Educational interventions included team-based approaches and lectures, and improved outcomes including vaccine knowledge, vaccination schedule, and cancer knowledge. Medical students with lower knowledge of HPV were more hesitant to recommend vaccination at baseline but were more likely to recommend vaccination after an education session.

**Discussion:**

Across HPS, inadequacies persist in HPV vaccine uptake, knowledge, and counseling comfort. It is critical to target vaccine uptake in this population and improve existing educational efforts to reduce preventable cancers. Institutions must prioritize HPV vaccine education to impact HPV related death.

## Introduction

In the US approximately 36,500 preventable cancers attributed to HPV are diagnosed yearly (1). Worldwide, 690,000 cases of cancers attributed to HPV are diagnosed each year (2). The most common HPV related cancer in women is cervical cancer, and in men is oropharyngeal cancer (3). While the incidence of cervical cancer has decreased over the past 15 years, there has been a significant increase in incidence rates of oropharyngeal, anal/rectal, and vulvar cancers (4).

HPV vaccination, first approved by the FDA in 2006 (5), is a safe and effective method to prevent HPV related cancers, and has substantially reduced the risk of invasive cervical cancer (6). The advisory council on immunization practices recommends routine HPV vaccination between ages 9-12, and catch up vaccination up to age 26 (7). For adults age 27-45, shared decision making is recommended to determine if a patient would benefit due to an elevated risk of HPV (7).

Unfortunately, the nation fell well below the Healthy People 2020 goal to achieve a HPV vaccination rate of 80%, with only 53% of females and 46% of males between 13-17 years up to date on HPV vaccination (8, 9). HPV vaccination is paramount to cancer prevention strategies. However, despite evidence of HPV vaccine safety, the rate of those citing safety concerns as the reason to not initiate the HPV vaccine series has increased 79.8% between 2015 and 2018 even though the rate of adverse events reported decreased to 29.4% in the same period (10). While evidence shows that the HPV vaccine is safe, patients continue to decline vaccines for reasons not backed in evidence including a belief that HPV vaccines are only for girls, should not be initiated prior to sexual debut, and is only approved up to age 15 (11). Myths and misinformation surrounding the HPV vaccine have been prevalent and active debunking of these myths by healthcare providers is critical (12–14).

To increase HPV vaccination rates, education surrounding HPV vaccine must be improved for healthcare providers in all fields. HPV vaccines are the only cancer prevention vaccine currently available, and it is a responsibility of all healthcare providers to recommend the vaccine and utilize all opportunities to vaccinate patients. With an intensification in vaccine hesitancy, it is important to confidently ensure parents that HPV vaccination is safe (10). Strong recommendation for HPV vaccine by a healthcare provider has been positively associated with vaccine uptake (15). HPV vaccination can be obtained in a variety of settings beyond the doctor’s office including schools (nurses) (16), pharmacies (17–19), and dental offices (20, 21). Efforts to increase HPV vaccine uptake must be recognized as an interprofessional mission by empowering HPS in a variety of professions to become vaccine champions. However, low fund of knowledge contributes to lack of confidence in recommending HPV vaccination across professions (19, 22–26). Additionally, previous work has shown that HPS who are vaccinated against HPV are significantly more likely to recommend HPV vaccines to their patients (27).

HPS must receive comprehensive education on HPV and HPV vaccines while in the formal learning environment. While several single-institution studies have assessed HPV knowledge and performed associated educational interventions, the efficacy and merits of these interventions have not been weighed against one another. Previous systematic reviews have synthesized educational interventions surrounding HPV vaccines in practicing healthcare providers, but not in HPS (24). The objective of our study was to evaluate the uptake of HPV vaccines, current HPV vaccine education initiatives, and HPV vaccine knowledge deficits in HPS.

## Methods

### Search strategy

The Preferred Reporting Items for Systematic Reviews and Meta-analyses (PRISMA) reporting guideline was used as a framework to guide the design and reporting of our study (28). We performed a systematic literature search from January 1, 2006, to July 21, 2021, to identify studies using the following databases: PubMed, Embase, Scopus, Web of Science, CINAHL. Articles prior to 2006 were not included because the HPV vaccine was not available. A previous systematic review on HPV knowledge among providers was reviewed for search term ideas (24). The complete search strategy is reported in **Table 1**.

**Table 1:** Literature search.

Database	Search terms	Filters	Results
**Pubmed**	("human papillomavirus" OR "human papilloma virus" OR HPV OR Alphapapillomavirus* OR “Wart virus*” OR Alphapapillomavirus[MeSH Terms]) AND (vaccin* OR Gardasil OR “Papillomavirus Vaccines"[MeSH Terms] OR Vaccine[MeSH Terms:NoExp]) AND (Student* OR School* OR Educat* OR Trainee* OR “Education, Medical, Undergraduate”[MeSH Terms] OR “Education, Nursing”[MeSH Terms] OR “Education, Dental”[MeSH Terms] OR “schools, medical”[MeSH Terms] OR “schools, dental”[MeSH Terms] OR “schools, pharmacy”[MeSH Terms] OR “schools, public health”[MeSH Terms] OR “schools, nursing”[MeSH Terms] OR “students, health occupations”[MeSH Terms]) AND (medical[Title/Abstract] OR nurs*[Title/Abstract] OR "public health"[Title/Abstract] OR dent*[Title/Abstract] OR pharm*[Title/Abstract] OR “physician assistant*”[Title/Abstract] OR "health occupation*"[Title/Abstract] OR "health profession*"[Title/Abstract])	English, 2006 – present	1428
**Embase**	“human papillomavirus” OR "human papilloma virus" OR HPV OR “wart virus” OR Alphapapillomavirus* OR 'Alphapapillomavirus'/exp OR 'Wart virus'/exp AND vaccin*.af. OR gardasil.af. OR 'wart virus vaccine'/exp OR 'wart virus vaccine' OR 'vaccine'/exp OR 'vaccine' AND Student* OR School* OR Educat* OR Trainee* OR 'medical student'/exp OR 'medical education'/exp OR 'nursing education'/exp OR 'physician assistant'/exp OR 'pharmacy student'/exp OR 'dental student'/exp OR 'dental education'/exp OR 'public health student'/exp AND medical:ti,ab,kw OR nurs*:ti,ab,kw OR 'public health':ti,ab,kw OR dent*:ti,ab,kw OR pharm*:ti,ab,kw OR 'physician assistant*':ti,ab,kw OR 'health occupation*':ti,ab,kw OR 'health profession*':ti,ab,kw	English, 2006 – present	2124
**Scopus**	TITLE-ABS-KEY("human papillomavirus" OR "human papilloma virus" OR HPV OR Alphapapillomavirus* OR “wart virus*”) AND TITLE-ABS-KEY(Vaccin* OR Gardasil OR “Wart virus vaccin*”) AND TITLE-ABS-KEY(Student* OR School* OR Educat* OR Trainee*) AND TITLE-ABS-KEY(medical OR nurs* OR "public health" OR dent* OR pharm* OR "physician assistant*" OR "health occupation*" OR "health profession*")	English, 2006 – present	2001
**CINAHL**	("human papilloma virus" OR HPV OR Alphapapillomavirus* OR “Wart virus*” OR Papillomavir* OR (MH "Papillomaviruses")) AND (Vaccin* OR Gardasil OR (MH "Papillomavirus Vaccine") OR (MH "Vaccines")) AND ((Student* OR School* OR Educat* OR Trainee*) OR (MH "Education, Medical+") OR (MH "Education, Nursing+") OR (MH "Education, Dental") OR (MH "Students, Physician Assistant") OR (MH "Students, Medical") OR (MH "Students, Dental") OR (MH "Students, Nursing+") OR (MH "Students, Pharmacy") OR (MH “schools, medical”) OR (MH “schools, dental”) OR (MH “schools, pharmacy”) OR (MH “schools, nursing”)) AND (TI medical OR TI nurs*" OR TI "public health" OR TI dent* OR TI pharm* OR TI "physician assistant*" OR TI "health occupation*" OR TI "health profession*" OR AB medical OR AB nurs*" OR AB "public health" OR AB dent* OR AB pharm* OR AB "physician assistant*" OR AB "health occupation*" OR AB "health profession*")	English, 2006 – present	237
**Web of Science**	TI=("human papillomavirus" OR "human papilloma virus" OR HPV OR Alphapapillomavirus* OR "Wart virus*" OR "Papillomavir*") OR AB=("human papillomavirus" OR "human papilloma virus" OR HPV OR Alphapapillomavirus* OR "Wart virus*" OR "Papillomavir*") AND TI=(Vaccin* OR Gardasil) OR AB=(Vaccin* OR Gardasil) AND TI=(Student* OR School* OR Educat* OR Trainee*) OR AB=(Student* OR School* OR Educat* OR Trainee*) AND TI=(medical OR nurs* OR “public health" OR dent* OR pharm* OR "physician assistant*" OR "health occupation*" OR "health profession*" OR AB=(medical OR nurs* OR “public health" OR dent* OR pharm* OR "physician assistant*" OR "health occupation*" OR "health profession*")	English, 2006 – present	895

Following the completion of searches, all articles identified were exported into EndNote and duplicates were removed. Articles were then imported into Covidence. First, titles and abstracts were reviewed independently by two reviewers (ML, LF, MH). Next, full text articles were obtained and reviewed by two reviewers. Following this, articles were screened for risk of bias by two reviewers to assess methodologic quality. Disagreements between reviewers were resolved by the third reviewer. Finally, data extraction was performed in covidence by two reviewers. A third reviewer compared results of data extraction by both reviewers to compile the final data which was exported for analysis.

For this review, HPS were defined as students in health professions that prescribe or promote HPV vaccination in their practice including medicine, nursing, pharmacy, public health, dentistry, and physician assistant. In studies that included practicing providers with students, >50% of the participants had to be students for inclusion. Inclusion criteria were as follows: 1) Original articles published in a peer-reviewed English language journals including quantitative, experimental, qualitative, quasi-experimental and observational studies. 2) Students attending school in the US due to the heterogeneity of health professional education worldwide. 3) Articles published in 2006 or later. 4) Studies with an objective to evaluate HPV vaccine uptake in HPS, knowledge of HPV vaccines in HPS, an educational intervention to improve knowledge of HPV vaccines in HPS, and/or HPS comfort counseling about HPV vaccination. Comfort counseling was defined as self-reported increase in confidence in counseling patients and/or their parents about HPV vaccination. The exclusion criteria were as follows: 1) Articles without full text, commentaries, editorials, perspective pieces, conference abstracts. 2) Studies of practicing healthcare providers including residents, fellows, nurses, dentists, pharmacists, physician assistants. Residents and fellows were excluded to focus on students in undergraduate medical education. 3) Studies of undergraduate students who were not in differentiated professional degree programs such as nursing.

### Data and analysis

The risk of bias was assessed with the Joanna Briggs Institute Tool for Cross Sectional Studies (29). The results of the risk of bias screening appear in **Table 2**. This tool encompasses inclusion criteria, whether the study subjects and setting were described in detail, exposure measurement in a valid and reliable way, confounding factors, strategies to deal with confounding factors, outcome measurement in a valid and reliable way, and appropriate use of statistics. Full text papers to be included were assessed for risk of bias by two independent reviewers by appraising the papers based on this criterion. Disagreements between reviewers were resolved by the third reviewer. All articles included in the study were reviewed based on this process.

**Table 2:** Risk of Bias Evaluation (30).

Name & Year	Were the study subjects and the setting described in detail?	Was the exposure measured in a valid and reliable way?	Were objective, standard criteria used for measurement of the condition?	Were confounding factors identified?	Were strategies to deal with confounding factors stated?	Were the outcomes measured in a valid and reliable way?	Was appropriate statistical analysis used?	Overall appraisal
Laitman 2018 (25)	Yes	Yes	Yes	No	No	Yes	Yes	Include
Afonso 2017 (31)	Yes	Yes	Yes	No	No	Yes	Yes	Include
Kepka 2019 (32)	Yes	Yes	Yes	No	No	Yes	Yes	Include
Laitman 2020 (33)	Yes	Yes	Yes	No	No	Yes	Yes	Include
Berenson 2017 (27)	Yes	Yes	Yes	No	No	Yes	Yes	Include
Wiley 2019 (22)	Yes	Yes	Yes	No	No	Yes	Yes	Include
Tsau 2011 (34)	Yes	Yes	Yes	No	No	Yes	Yes	Include
Wiley 2019 (22)	Yes	Yes	Yes	No	No	Yes	Yes	Include
Guadiana 2021 (20)	Yes	Yes	Yes	No	No	Yes	Yes	Include
Rutkoski 2020 (35)	Yes	Yes	Yes	No	No	Yes	Yes	Include
Schnaith 2018 (36)	Yes	Yes	Yes	No	No	Yes	Yes	Include
Cotter 2020 (37)	Yes	Yes	Yes	No	No	Yes	Yes	Include
Wiley 2018 (30)	Yes	Yes	Yes	No	No	Yes	Yes	Include
Hollins 2021 (38)	Yes	Yes	Yes	No	No	Yes	Yes	Include
Evans 2020 (39)	Yes	Yes	Yes	No	No	Yes	Yes	Include
Walker 2018 (40)	Yes	Yes	Yes	No	No	Yes	Yes	Include
Daniel 2021 (17)	Yes	Yes	Yes	No	No	Yes	Yes	Include
Torres 2020 (26)	Yes	Yes	Yes	No	No	Yes	Yes	Include
Berenson 2020 (41)	Yes	Yes	Yes	No	No	Yes	Yes	Include
Berenson 2015 (42)	Yes	Yes	Yes	No	No	Yes	Yes	Include
Berenson 2021 (43)	Yes	Yes	Yes	No	No	Yes	Yes	Include

Studies selected for inclusion had data extracted by two independent reviewers. The Template for Intervention Description and Replication (TIDiEr) checklist was used to develop the data collection for HPV knowledge interventions (44).

## Results

### Literature search

**Figure 1:**
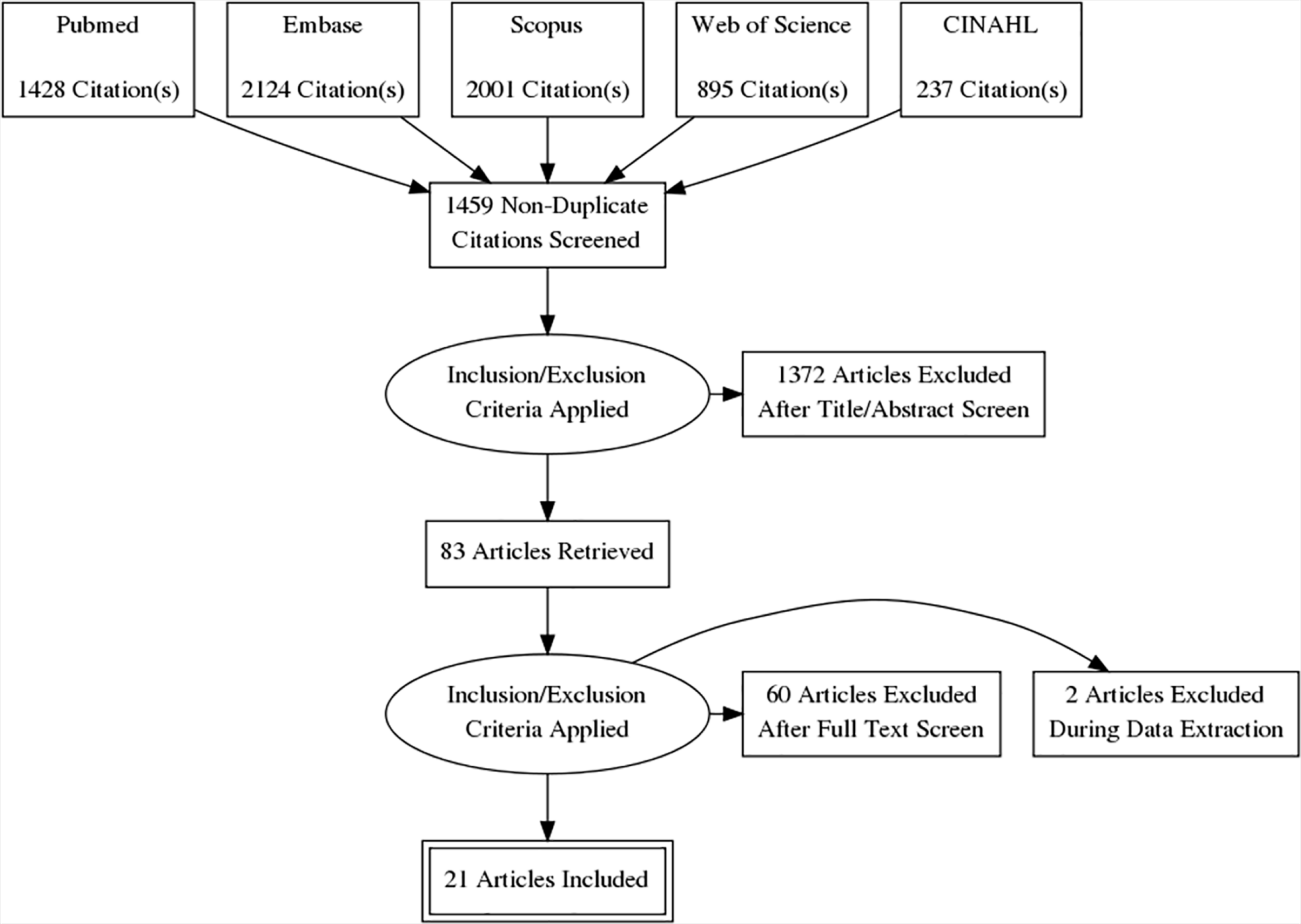
PRISMA diagram.

A full breakdown of the literature search appears in a PRISMA diagram (**Figure 1**). The initial search contained 6684 articles. 5206 duplicates were removed in EndNote. 1459 studies were screened by title and abstract, and 1372 studies were eliminated based on inclusion and exclusion criteria. Full-text review was performed for 83 articles. Sixty articles were excluded because the study did not take place in the US (n = 46), not a student population (n = 7), covered other vaccines (n = 3), did not have full text available (n = 2), duplicate (n = 1), not a health profession included in the study (n = 1), undergraduate students (n = 1), or measured the wrong outcome (n = 1). An additional 2 articles were excluded during data extraction.

A total of 21 articles were included in the analysis (**Table 3**) (20, 22, 25–27, 30–43, 45, 46). Of the studies included, 20 included knowledge data, 11 included vaccine uptake, 8 included interventions, and 12 included counseling comfort. The students in the studies included medical (n=14), dental (n=7), dental hygiene (n=6), nursing (n=3), physician assistant (n=2), public health (n=1), and pharmacy (n=1). Most participants across all studies that reported gender were female (56.3%).

**Table 3:** Demographics.

Name & year	Title	Student types	Region	N	Male	Female	Age
Laitman 2018 (25)	Medical Student Knowledge of Human Papillomavirus Positive Head and Neck Cancer	Medical	Northeast	617	Not reported	Not reported	Not reported
Afonso 2017 (31)	Will they lead by example? Assessment of vaccination rates and attitudes to human papilloma virus in millennial medical students	Medical	Midwest	214	101	112	<25 (N=141); 26-30 (N=64); 31 (N=8)
Kepka 2019 (32)	US oral health students' willingness to train and administer the HPV vaccine in dental practices	Dental, Dental Hygiene	Other	306	91	215	18-29 (N=245); 61 not reported
Laitman 2020 (33)	US Medical Trainees' Knowledge of Human Papilloma Virus and Head and Neck Cancer	Medical	Other	2046	746	1300	Not reported
Berenson 2017 (27)	US medical students' willingness to offer the HPV vaccine by vaccination status	Medical	South	231	109	122	<25 (N = 163); 26 (N = 68)
Wiley 2019 (22)	Team-Based Learning Module for Undergraduate Medical Education: A Module Focused on the Human Papilloma Virus to Increase Willingness to Vaccinate	Medical	South	896	Not reported	Not reported	20-25 years old (N=887)
Tsau 2011 (34)	The knowledge levels and opinions of biomedical students regarding the human papillomavirus quadrivalent (types 6, 11, 16, and 18) recombinant vaccine	Medical, Pharmacy, Physician Assistant	Midwest	868	403	716	Mean age = 24.51
Wiley 2019 (22)	Relationship Between Intent to Vaccinate and the Education and Knowledge of Human Papillomavirus Among Medical School Faculty and Students in Texas	Medical	South	895	372	523	<30 (N=842)
Guadiana 2021 (20)	Oral health care professionals recommending and administering the HPV vaccine: Understanding the strengths and assessing the barriers	Dental, Dental Hygiene	Midwest	392	128	265	Dental students mean age= 25.1 (N= 239) Dental Hygiene students mean age = 24.2 (n=150)
Rutkoski 2020 (35)	A Multi-state Evaluation of Oral Health Students' Knowledge of Human Papillomavirus-Related Oropharyngeal Cancer and HPV Vaccination	Dental, Dental Hygiene	Other	359	107	258	18-29 (N=291); 30+ (N=78)
Schnaith 2018 (36)	An innovative medical school curriculum to address human papillomavirus vaccine hesitancy	Medical	Midwest	101	33	67	Not reported
Cotter 2020 (37)	Impact of HPV Immunization Training on Dental Hygiene Students' Attitudes and Confidence Regarding HPV Preventive Education	Dental Hygiene	South	40	Not reported	Not reported	Not reported
Wiley 2018 (30)	Human Papillomavirus: From Basic Science to Clinical Management for Preclinical Medical Students	Medical	South	243	Not reported	Not reported	Not reported
Hollins 2021 (38)	Human Papillomavirus Vaccination Status and Parental Endorsement Intentions among Undergraduate Student Nurses	Nursing	South	153	Not reported	Not reported	<26 (N=112); 26 (N=41)
Evans 2020 (39)	HPV Knowledge and Attitudes Among Medical and Professional Students at a Nevada University: A Focus on Oropharyngeal Cancer and Mandating the Vaccine	Medical, Physician Assistant, Public Health	West	221	88	132	19-26 (N=121); 27 (N=79)
Walker 2018 (40)	HPV and Oral Cancer: The Need to Integrate Oral Health Practices into Nursing Education	Dental, Dental Hygiene, Nursing	Midwest	99	35	123	18-19 (N= 3); 20-24 (N= 48); 25-29 (N= 50); 30-39 (N= 42); 40: (N= 14)
Daniel 2021 (17)	HPV and HPV Vaccination Knowledge and Attitudes Among Medical Students in Alabama	Medical	South	127	65	62	18-26 (N= 106); 26 (N=21)
Torres 2020 (26)	Assessing Dental Students' HPV Health Literacy and Intention to Engage in HPV-Related Oropharyngeal Cancer Prevention	Dental	South	109	43	66	25.79 (mean)
Berenson 2020 (41)	An educational intervention to improve attitudes regarding HPV vaccination and comfort with counseling among US medical students	Medical	South	256	143	113	<30 (N = 242); 30 (N = 14)
Berenson 2015 (42)	A brief educational intervention increases providers' human papillomavirus vaccine knowledge	Medical	South	208	135	286	<30 (N=317); 30-49 (N=62); 50 (N=41), Unknown (N=7)
Berenson 2021 (43)	A brief educational intervention can improve nursing students' knowledge of the human papillomavirus vaccine and readiness to counsel	Medical, Nursing	South	900	334	553	<30 (N=837) 30 and above (N=54)

### Vaccine uptake

**Table 4** summarizes the vaccine uptake of participants in all studies that included this measure. Most participants were under age 30, and therefore eligible for HPV vaccination during childhood. Across studies, the rate of reported HPV vaccine series initiation ranged from 34.6% to 70.3%, and the rate of those up to date on HPV vaccination ranged from 28.3% to 58.3%. Multiple studies noted that highest rate of vaccine uptake was in participants under age 25 (27, 31, 46). In studies that reported vaccination rate by gender, female students were more likely to be vaccinated (27, 31, 46). Provider recommendation also influenced the decision to get the HPV vaccine in multiple studies (38, 47).

**Table 4:** Vaccine uptake.

Name & year	Student types	N	Students who have initiated HPV vaccine series	Students who have not initiated HPV vaccine series	Students up to date on HPV vaccine	Students who are not up to date on HPV vaccination
Afonso 2017 (31)	Medical	214	94 (43.9%)	119 (55.6%)	75 (35.0%)	138 (64.5%)
Berenson 2017 (27)	Medical	231	97 (42.0%)	134 (58.0%)	79 (34.2%)	152 (65.8%)
Wiley 2019 (22)	Medical	842	Not reported	Not reported	453 (53.8%)	Not reported
Schnaith 2018 (36)	Medical	101	71	28	71	28
Hollins 2021 (38)	Nursing	153	89 (58.2%)	64 (41.8%)	65 (42.5%)	88 (57.5%)
Mann 2020 (47)	Dental	293	Not reported	Not reported	110 (37.5%)	183 (62.4%)
Evans 2020 (39)	Medical, Physician Assistant, Public Health	221	122 (55.2%)	99 (44.8%)	99 (44.8%)	122 (55.2%)
Daniel 2021 (17)	Medical	127	44 (34.6%)	74 (58.3%)	36 (28.3%)	82 (64.6%)
Torres 2020 (26)	Dental	109	67 (61.5%)	42 (38.5%)	33 (30.3%)	60 (55.0%)
Berenson 2020 (41)	Medical	256	114 (44.5%)	142 (55.5%)	Not reported	Not reported
Berenson 2021 (43)	Medical, Nursing	900	455 (50.6%)	294 (32.7%)	Not reported	Not reported

### Knowledge

**Table 5** describes the level of HPV knowledge among HPS. Knowledge gaps surrounding the link between HPV and head and neck cancers were most notable. Laitman et al. reported in two studies that 99% (611/617) and 99.4% (2033/2046) of medical students correctly identified HPV infection as a cause of cervical cancer, but only 47.2% (291/617) and 40.3% (825/2046) correctly connected HPV with head and neck cancers (25, 33). Medical, physician assistant and public health students demonstrated a similar knowledge gap regarding HPV and oropharyngeal cancers. Evans et al. found that only 45% (90/200) of medical students knew that HPV could cause oropharyngeal cancer (39). Overall, dental and dental hygiene students had greater understanding of the link between HPV and oropharyngeal cancer, ranging from 97.3% to 70% (26, 37). However, Walker et al. found that only 5.1% (8/158) of dental, dental hygiene, and nursing students knew that HPV vaccines may be effective in protecting against HPV and related oropharyngeal cancer (40).

**Table 5:** Knowledge.

No./total (%)				
Author and year	HPV causes cervical cancer	HPV related cancers (correctly identified whether HPV causes this cancer)	HPV vaccine is safe	Additional results
Laitman 2018 (25)	611/617 (99.0)	Anal Cancer: 473/617 (76.7)Genital Cancer: 468/617 (75.9)Head and Neck Cancer: 291/617 (47.2)Esophageal cancer: 340/617 (55.1)		Fewer students knew that HPV could lead to recurrent respiratory papillomatosis or head and neck cancer than knew it can lead to cervical cancer
Afonso 2017 (31)	195/214 (91.1)	Cancers that are not cervical cancer: 105/214 (49.1)	191/214 (89.3)	- 44 (21%) did not know that the vaccine was recommended for girls and boys. - Fully vaccinated participants scored better in knowledge items than non-vaccinated participants. - Female participants scored better than males.
Kepka 2019 (32)		Oropharyngeal cancer: 286/306 (93.5)		
Laitman 2020 (33)	2033/2046 (99.4)	Head and neck cancer: 825/2046 (40.3)Esophageal cancer: 885/2046 (43.3)		
Berenson 2017 (27)	196/223 (87.9)		212/222 (95.5)	Genital warts are caused by the same HPV types that cause cervical cancer: 176/228 (77.2)
Wiley 2019 (22)				First year medical students in the intervention population scored significantly higher than graduating seniors across the state in vaccine knowledge (intervention group = 91.9%, statewide = 81.8%, P .001).
Tsau 2011 (34)	974/1002 (97.2)	Prostate cancer: 853/1002 (85.1)	751/1002 (75.0)	Women (n = 673) scored higher than men (n = 328) on the total knowledge test (76% vs 68%).First year students had lower knowledge scores than second year students (70.7% vs 75%)
Wiley 2019 (22)				- Students scored better than faculty regarding general knowledge *(p* = .0019) but not regarding vaccine-related knowledge - Clinical students had higher general knowledge scores than either preclinical faculty or students - Clinical students had higher general knowledge scores than clinical faculty
Rutkoski 2020 (35)	353/380 (92.9)	- What percentage of oropharyngeal cancer is attributed to HPV? 46/380 (12.1) - HPV vaccines can protect men and women against HPV related oropharyngeal cancer: 289/380 (76.1) - HPV vaccines can protect men and women against HPV related anal cancer: 209/380 (55.0) - HPV can cause oropharyngeal cancer: 289/380 (76.0)	3452/380 (90.0)	- Male participants, participants who were 30 years and older, participants whose program was in Tennessee, and participants whose program was in a conservative region of the U.S. had lower proportions of students with adequate HPV vaccination knowledge.
Cotter 2020 (37)		Anal cancer. Pre-test: 21/37 (56.8); Post-test: 37/37 (100)Oral cancer. Pre-test: 36/37 (97.3); Post-test: 37/37 (100)		HPV causes cancer. Pre-test: 34/37 (91.9); Post-test: 37/37 (100)
Wiley 2018 (30)				- Students improved their total HPV-related knowledge score after the intervention from 66.3% to 86.3%. - Students improved their general HPV-related knowledge score from 58.4% to 83.2% after the intervention.
Evans 2020 (39)		Oropharyngeal cancer: 90/200 (45.0)	198/200 (99.0)	Knowledge check Pre and Post-Test of just MS1 and MS2 students (120 students): What cancers can be caused by HPV?Average Pre-Test: 3.53/5Average Post-Test: 4.67/5
Walker 2018 (40)		Oropharyngeal cancer: 8/158 (5.1)		More nursing (90%) and nurse practitioner students (97%) than dental (78%) and dental hygiene students (83%) knew that the rise in oral and oropharyngeal cancer in Caucasian people is related to HPV infection.
Daniel 2021 (17)		Ovarian cancer: 56/117 (47.9)Prostate cancer: 74/117 (63.2)Anal cancer: 87/117 (74.4)Penile cancer: 88/117 (75.2)Oropharyngeal cancer: 90/117 (76.9)	115/117 (98.3)	Program year had the largest observed effect with more advanced students scoring higher HPV knowledge scores (*p* 0.0001) and higher HPV vaccination knowledge scores (*p* = .0069).
Torres 2020 (26)	90/109 (82.6)			Roughly 90% of cervical, anal, and vulvar cancers are caused by high-risk HPV infections: 90/109 (82.6)Roughly 70% of oropharyngeal cancer is caused by high-risk HPV infections: 85/109 (78.0%)
Berenson 2020 (41)				The greatest increase in knowledge was among those correctly reporting the follow-up dosing schedule for patients ≥15 years old.The proportion of participants who answered incorrectly about restarting the three-dose series decreased 82% between pre and post-test.
Berenson 2015 (42)		HPV vaccine prevents cancer of: Vulva: Pre-Test: 78/208 (37.5) Post-Test: 182/208 (87.5)Vagina: Pre-Test: 66/208 (31.7) Post-Test: 181/208 (87.0)Anus: Pre-Test: 125/208 (60.1) Post-Test: 192/208 (92.3)Penis: Pre-Test: 98/208 (47.1) Post-Test: 182/208 (87.5)Ovaries:. Pre-test: 197/208 (94.7); Post-test: 192/208 (92.3)		Medical students had greater baseline knowledge than other healthcare workers about the types of cancer prevented by the HPV vaccine.Female had higher baseline knowledge than males (*p*=.023)The mean knowledge score went up to 14.7 (SD=1.9) after the education intervention.
Berenson 2021 (43)				Nursing students had lower baseline scores than medical students and were less familiar with HPV vaccination guidelines.

HPS also demonstrated knowledge gaps in the vaccine dosing schedule. Berenson et al. found that only 25.1% (44/175) of medical students knew whether a patient should restart the vaccine series if their last dose was more than a year ago (27). Cotter et al. demonstrated that only 35.1% (13/37) and 37.8% (14/37) of dental hygiene students identified the correct number of doses for those under age 15 and those at or over age 15 respectively (37). Berenson et al. found that only 31.3% (80/256) of medical students knew the CDC recommended schedule of follow up doses for those 15 years or older (41).

The majority of students in each study knew that the HPV vaccine is safe, though there was a wide range between 75% (751/1002) and 99% (198/200) of HPS with this knowledge (34, 39).

### Educational interventions

**Table 6** summarizes the key findings of the studies regarding educational interventions. Most interventions combined lectures and group activities. Topics covered included HPV virology, HPV-related cancers, epidemiology, vaccines, and comfort counseling. All studies reported improved knowledge following educational interventions (36, 37, 39, 41, 42, 46).

**Table 6:** Interventions.

Author and year	Student type	Setting	Intervention model	Outcome	Recommendations
Wiley 2019 (22)	Medical	Preclinical lecture series	Lecture series, group activities, pre and post test	- HPV knowledge - Willingness to recommend HPV vaccination to patients	Medical schools should include modules to improve knowledge of HPV vaccination.
Schnaith 2018 (36)	Medical	Preclinical lecture, video, and role play	Lecture series, role play	- Awareness of benefits of HPV vaccine - Likelihood of recommending the vaccine - Comfort counseling	Medical schools should introduce module to increase student awareness of HPV vaccine benefits.
Cotter 2020 (37)	Dental hygiene	Junior and Senior student education module	Education module, pre and post test	- Knowledge of HPV - Knowledge of HPV vaccination - Comfort counseling	Dental hygiene schools should include educational modules to increase knowledge and confidence in providing HPV and HPV vaccine education.
Wiley 2018 (30)	Medical	Preclinical module	Week-long module, group exercises, quizzes	- Knowledge of HPV - Satisfaction of HPV - Vaccine recommendation	Medical schools should include modules to improve knowledge of HPV vaccination
Evans 2020 (39)	Medical, physician assistant, public health	Survey and workshop	Vaccine workshop	- Knowledge of HPV associated malignancies.	Health professions schools with multiple types of students should consider an interprofessional HPV workshop to increase knowledge of HPV related cancers.
Berenson 2020 (41)	Medical	Lectures in scheduled clerkships	Presentation and survey	- Attitudes toward the HPV vaccine. - Comfort with counseling families.	Material on HPV vaccination should be included in medical student clerkship curriculums.
Berenson 2015 (42)	Medical	Lectures	Lectures and surveys	- Knowledge of HPV. - Comfort with counseling families.	Medical students should have lectures on HPV epidemiology and the HPV vaccine to improve knowledge, and the quality of their vaccine counseling.
Berenson 2021 (43)	Medical, Nursing	Structured presentations	Presentations	- Attitudes toward the HPV vaccine. - Comfort with counseling families.	Medical and nursing students should have lectures about HPV vaccination to improve attitudes toward the HPV vaccine and comfort with counseling families.

Wiley et al. demonstrated that a team-based instructional module with video simulations and clinical vignettes improved HPV and vaccine knowledge among first year medical students (46). Students in the intervention group showed significant general knowledge improvement (from 58.4% to 85.3% P<.001) and vaccine-related knowledge (from 78.8% to 91.7% P<.001). In another study by Schnaith et al. (2018), the intervention consisted of a lecture, video and role-play simulation (36). The intervention increased awareness of the benefits of the HPV vaccine by 0.82 points on a Likert scale of 1-5.

In Berenson et al. (2015), a brief lecture presentation discussing HPV and vaccination for healthcare workers significantly improved HPV knowledge (42). Medical students participating in a 12 lecture series covering HPV and vaccination in Berenson et al., 2020 demonstrated significant improvement in knowledge and comfort counseling patients regarding HPV vaccination (41). In Berenson et al. (2021), a similar intervention was presented to nursing students covering dosing schedules, efficacy and safety of the vaccine with similar improvement in knowledge scores (43).

In Evans et al. (2020), first- and second-year medical students attended a 1.5-hour HPV vaccine workshop covering HPV and strategies to address vaccine hesitancy (39). After the workshop, students demonstrated improved scores in the area of HPV-related malignancies (mean improved from 3.53 to 4.67), transmission (3.05 to 3.87), symptoms (1.76 to 2.63), and vaccination schedule (1.21 to 1.68).

In Cotter et al. (2020), an educational intervention for dental hygiene students consisted of a one-hour educational module covering the prevalence of HPV, HPV oropharyngeal cancers, HPV vaccination and information on counseling techniques (37). Following the intervention, the mean score on the knowledge test increased from 8.75 to 13.32 (scale 0-15).

These results demonstrate that a variety of effective educational strategies for improving knowledge about HPV and HPV vaccination. While elaborate interventions such as the team-based learning modules outlined in Wiley et al. (2018) and the three-part educational curriculum created in Schnaith et al. (2019) are effective, simpler models such as the one-hour educational module discussed in Cotter et al. (2019) and the 1.5 hour workshop in Evans et al. (2020) can be effective, and may be easier for institutions to implement (31, 32, 36, 37, 39, 46–48).

### Counseling comfort

**Table 7** summarizes the key findings regarding HPS comfort recommending and discussing the HPV vaccine. Among medical students, Berenson et al. found that more vaccinated (85/93 91.4%) than unvaccinated (101/122 82.8%) students agreed that they were comfortable discussing the benefits and risk of HPV vaccination (27). However, Wiley et al. found no difference in willingness to recommend the vaccine between vaccinated and unvaccinated students (46).

**Table 7:** Counseling comfort.

Name & year	Student types	Confidence in recommending or counseling about HPV vaccination	Other findings
Wiley 2018 (30)	Medical 243	Medical students’ confidence recommending HPV vaccine to all *patients* increased on average 0.98. *(5-point Likert scale)*	
Wiley 2019 (22)	Medical 234	Medical students’ confidence recommending HPV vaccine to all *patients* increased on average 0.98.*(5-point Likert scale)*	• For each unit increase in general knowledge score, the odds of a student recommending HPV vaccination increased 10%.
Berenson 2017 (27)	Medical 231	86.5% of medical students (186/215) would recommend the HPV vaccine to *patients*. 88.0% of medical students (191/217) would recommend the HPV vaccine to *parents*.	•Unvaccinated students were more likely than vaccinated students to delay recommendation of the HPV vaccine until patients are 15 or 16 years old • More vaccinated (91.4%) than unvaccinated (82.8%) students comfortable counseling patients.
Berenson 2020 (41)	Medical 256	Medical students’ confidence recommending HPV vaccine to *female patients* increased from 0.32 → 0.96. Medical students’ confidence recommending HPV vaccine to *male patients* increased from 0.25 → 0.95. Medical students’ confidence recommending HPV vaccine to *parents of girls* increased from 0.31 → 0.94. Medical students’ confidence recommending HPV vaccine to *parents of boys* increased from 0.28 → 0.95.*(Scale of 0-1)*	• Comfort counseling males about the HPV vaccine improved the most post-intervention. • Much of the increased comfort occurred among Asian and Hispanic students. • A significant increase in counseling comfort occurred post-intervention among students who had not received the HPV vaccine.
Berenson 2021 (43)	Medical 388 Nursing 512	***Medical students*** Pre intervention 49.8% (255/512) would recommend the HPV vaccine to *women* compared to 93.0% (476/512) after the intervention. Pre intervention 46.1% (236/512) would recommend the HPV vaccine to *men* compared to 92.8% (475/512) after the intervention. Pre intervention 49.8% (255/512) would recommend the HPV vaccine to *parents of girls* compared to 92.8% (475/512) after the intervention. Pre intervention 47.9% (245/512) would recommend the HPV vaccine to *parents of boys* compared to 92.6% (474/512) after the intervention. ***Nursing students*** Pre intervention 39.2% (152/388) would recommend the HPV vaccine to *women* compared to 86.9% (337/388) after the intervention. Pre intervention 37.4% (145/388) would recommend the HPV vaccine to *men* compared to 85.1% (330/388) after the intervention. Pre intervention 39.9% (155/388) would recommend the HPV vaccine to *parents of girls* compared to 86.9% (337/388) after the intervention. Pre intervention 39.9% (155/388) would recommend the HPV vaccine to *parents of boys* compared to 84.5% (328/388) after the intervention.	• Post-intervention, more nursing students than medical students still needed education to counsel patients about HPV vaccination • More students were comfortable educating vaccine-hesitant patients' post-intervention
Afonso 2017 (31)	Medical 214	40.2% of medical students (86/214) would recommend the HPV vaccine to *patients*.	• No difference between male students' and female students' comfort counseling.
Schnaith 2018 (36)	Medical 101	Medical students’ confidence recommending HPV vaccine to all *patients* increased on average increased on average 1.37. *(5-point Likert scale)*	
Daniel 2021 (17)	Medical 127	47.4% of medical students (54/114) would recommend the HPV vaccine to *patients*. 70.4% of medical students (81/115) would recommend the HPV vaccine to *parents*.	• More advanced-year students demonstrated more positive attitudes toward HPV vaccination (*p*=.0003).
Kepka 2019 (32)	Dental 73 Dental hygiene students 233		• Many dental and dental hygiene students did not perceive that it was the role and scope of an oral health professional to recommend or administer the HPV vaccine.
Guadiana 2021 (20)	Dental 150 Dental hygiene242	Dental students’ confidence recommending HPV vaccine to all *patients* was 3.4 on average. *(5-point Likert scale)* Dental hygiene students’ confidence recommending HPV vaccine to all *patients* was 3.2 on average. *(5-point Likert scale)*	• Stronger confidence discussing HPV with patients and stronger beliefs that the vaccine enhances patients’ health were positively associated with willingness to administer the vaccine. • Increasing confidence discussing HPV by one point (5-point scale) increased the odds of willingness to administer the vaccine by a factor of 1.30 (95% CI, 1.04–1.61). • A one-point increase in the belief that the vaccine enhances patients’ health increased odds of willingness to administer the vaccine by a factor of 1.48 (95% CI, 1.21–1.81).
Cotter 2020 (37)	Dental hygiene 40	Dental hygiene students’ confidence recommending HPV vaccine to all *patients* increased by 0.48 on average. *(5-point Likert scale)* Dental hygiene students’ confidence recommending HPV vaccine to *parents* increased by 0.46 on average. *(5-point Likert scale)*	
Walker 2018 (40)	Nurse practitioner 58 Dental students 29 Nursing 12 Dental hygiene 59		• 84.2% (133/158) students felt it was within their scope of practice to advise about vaccination for oral cancer. • 79.1% (125/158) students felt it was within their scope of practice to advise about vaccination for all human papillomavirus cancers. • 89.9% (142/158) students felt it was within their scope of practice to counsel about the link between human papillomavirus and oral cancer.
Hollins 2021 (38)	Nursing 153		• There were no significant differences in adolescent or parent counseling skills satisfaction between vaccine non-initiators and initiators or between vaccine initiators and completers.

Wiley et al. found that medical students who were hesitant or ambivalent about recommending the HPV vaccine scored lower on the total HPV knowledge assessment (*p*=.026) and general HPV knowledge assessment (*p*=.03) compared to students who were likely to recommend vaccination (46). Similarly, Daniel et al. found that students further along in medical school demonstrated more positive attitudes toward HPV vaccination (*p*=.0003) (45).

Each study of medical students that included an educational intervention showed improvement in their ability to counsel others regarding HPV vaccination. Wiley et al. reported that 100% of students agreed that they would recommend the vaccine as a provider after a week-long team-based learning exercise (46). Schnaith et al. found an increase in students’ comfort conversing with HPV vaccine-hesitant parents/patients after an educational HPV curriculum (36). Berenson et al. reported significant increases in medical students’ comfort counseling parents and eligible men and women about the vaccine following HPV lectures (41). A significant increase in counseling comfort occurred among students who had not received the HPV vaccine. In another study, Berenson et al. demonstrated that medical and nursing students were significantly more comfortable educating vaccine-hesitant patients post-intervention (43). Generally, the studies found more hesitancy within vaccine counseling from non-medical HPS than medical students. Kepka et al. found that dental and dental hygiene students did not perceive that it was the role of an oral health professional to recommend or administer the HPV vaccine (32).

Studies among dental and dental hygiene students found that educational interventions improved comfort surrounding administering and discussing HPV vaccines. Guadiana et al. found that dental and dental hygiene students who were more confident in their ability to discuss HPV were more likely to be willing to administer the vaccine (OR 1.30, 95% CI 1.04–1.61) (20). Additionally, a one-point increase in the belief that the vaccine enhances patients’ health increased the students’ willingness to administer the vaccine (OR 1.48, 95% CI 1.21–1.81). Cotter et al. found that after an interactive online training module, dental hygiene students increased their confidence providing HPV vaccination counseling to patients (*p* =.033).

## Discussion

An estimated 79 million people in the United States are infected with HPV, and in individuals not able to clear the virus, leads to cancer. Despite significant data demonstrating HPV vaccination efficacy, vaccination rates remain stagnant; and HPV related oropharyngeal and anal/rectal cancers are on the rise, particularly among men (49). Our study systematically examines HPV vaccine uptake, knowledge, counseling comfort and interventions to increase HPV knowledge among HPS, the next generation of healthcare providers.

The rates of HPV vaccine series initiation across studies ranged from 34.6% to 70.3%, and the rate of those up to date on HPV vaccination ranged from 28.3% to 58.3% (27, 31, 46). Rates of vaccination were the highest among those under 25, and female (27, 31, 46). Moreover, provider recommendation of the vaccine influenced the decision to get vaccinated in multiple studies – emphasizing why educating students as the next generation of providers is so critical (38, 47). In order to achieve the goals of Healthy People 2030 and the World Health Organization (WHO) goal to eradicate cervical cancer by 2030, increasing the vaccination rate to 90% is essential (48). A strong healthcare workforce of HPV vaccine advocates is critical to this mission.

While students consistently displayed awareness that HPV causes cervical cancer (99%), many lacked knowledge of the ability of HPV to cause other cancers including head and neck cancer (40-47%) and oropharyngeal cancer (45%) (33, 40). Most students did know that HPV vaccination is safe (75-99%) (34, 39). Other knowledge gaps about HPV vaccination included dosing schedules, age ranges, boys needing vaccination, and whether to initiate the vaccine series prior to sexual activity. However, while rates of cervical cancer have decreased from improved screening and HPV vaccination, the US still lags behind other developed nations in their progress towards cervical cancer eradication (50).

Educational interventions varied in format but were consistently effective in increasing knowledge of HPV. One team-based instruction module significantly increased general HPV knowledge, HPV vaccine related knowledge, and likelihood of vaccine recommendation (46). A different workshop, which also covered vaccine hesitancy, significantly improved knowledge of HPV related cancers, symptoms, and vaccine dosing schedules (37). While many of the interventions were time intensive, the shorter interventions also increased knowledge. Finding a balance between length of intervention necessary to provide sufficient education but remain feasible to implement amongst the many topics covered in a medical education is essential.

Additionally, comfort counseling patients about HPV vaccination was low – but increased following specific interventions targeting this objective. Students across studies cited similar barriers to HPV vaccine uptake to their patients including parental decline of the vaccine, not being recommended the vaccine by a healthcare provider (27, 38, 45). Moreover, previous studies have shown that students who were vaccinated against HPV were more likely to recommend vaccination, especially to adolescents (27). Following educational interventions, more students felt comfortable recommending HPV vaccination. It is critical to target efforts to increase vaccination rates amongst students in the health professions. This should include availability of the HPV vaccine at school sponsored health services and ensuring that providers at these sites are educated about the importance of HPV vaccination. Additionally, since most health professional schools require flu vaccination for students each year, schools should consider offering HPV vaccination alongside flu vaccinations. HPV vaccination prevents not only HPV related cancers but also pre-invasive disease such as cervical dysplasia, which in women of reproductive age, can be quite devastating.

Limitations of this analysis include that most are single institutional studies and are thus subject to any bias resulting from the studies themselves. The risk of bias has been minimized through screening prior to inclusion. Additionally, it is of note that there are multiple studies from the same authors because there are investigators who have conducted multiple studies in this area.

Future studies should focus on ways to target vaccine uptake in HPS and expand upon existing knowledge interventions to implement better education on a broader scale. This education should utilize an interprofessional approach is vital to expanding the number of knowledgeable professionals counseling and offering HPV vaccination to prevent HPV-related cancers. The papers reviewed in this analysis demonstrated that various educational modalities of different lengths effectively increase knowledge of HPV and HPV vaccination. Further studies should focus on educational methods to help determine optimal approaches by objective measures such as a significant increase in HPV vaccination. Ultimately, we must take active efforts to improve the quality of education provided. Taking these steps to enhance HPS education is critical to lowering the number of missed opportunities for HPV vaccination in a variety of healthcare settings.

## Conclusion

Across HPS, major gaps persist in HPV vaccine uptake, knowledge, and counseling comfort. It is critical to target vaccine uptake in this population, including ensuring availability of vaccination for students. Educational institutions must make education about the HPV vaccine a priority in both preclinical and clinical curriculums. This study highlights the need for interventions to improve HPV vaccination uptake and education on HPV in HPS to train healthcare professionals that strongly recommend HPV vaccination to prevent cancer.

## Data availability statement

The original contributions presented in the study are included in the article/Supplementary Material. Further inquiries can be directed to the corresponding author.

## Author contributions

ML and MH had full access to all the data in the study and took responsibility for the integrity of the data and the accuracy of the data analysis. Concept and design, all authors. Acquisition, analysis, or interpretation of data, all authors. Drafting of the manuscript, ML, LF, and KL. Critical revision of the manuscript for important intellectual content, all authors. Statistical analysis, ML, LF, and KL. Administrative, technical, or material support, MH and PJ. Supervision, MH and PJ. All authors contributed to the article and approved the submitted version.

## Acknowledgments

The authors thank Dr. Barbara Sorondo and John Reynolds from Calder Library for consultation on study methodology.

## Conflict of interest

The authors declare that the research was conducted in the absence of any commercial or financial relationships that could be construed as a potential conflict of interest.

## Publisher’s note

All claims expressed in this article are solely those of the authors and do not necessarily represent those of their affiliated organizations, or those of the publisher, the editors and the reviewers. Any product that may be evaluated in this article, or claim that may be made by its manufacturer, is not guaranteed or endorsed by the publisher.
